# Notes on two closely related spider species of the *Pholcus phungiformes* species group (Araneae, Pholcidae) from Beijing, China

**DOI:** 10.3897/zookeys.965.56199

**Published:** 2020-09-03

**Authors:** Xiang Wang, Shumaila Shaheen, Qiaoqiao He, Zhiyuan Yao

**Affiliations:** 1 College of Life Science, Shenyang Normal University, Shenyang 110034, Liaoning, China Shenyang Normal University Shenyang China; 2 Liaoning Key Laboratory of Evolution and Biodiversity, Shenyang 110034, Liaoning, China Liaoning Key Laboratory of Evolution and Biodiversity Shenyang China; 3 Liaoning Key Laboratory for Biological Evolution and Agricultural Ecology, Shenyang 110034, Liaoning, China Liaoning Key Laboratory for Biological Evolution and Agricultural Ecology Shenyang China

**Keywords:** daddy-long-leg spider, DNA barcode, morphology, Pholcinae, taxonomy

## Abstract

The *Pholcus phungiformes* species group is highly diverse and currently contains 53 species. In this study, *Pholcus tongyaoi* Wang & Yao, **sp. nov.** (male, female) from Huairou District, Beijing, China is described while similar congener *Pholcus lexuancanhi* Yao, Pham & Li, 2012 from neighboring Haidian District (type locality) is redescribed; the female of *P. lexuancanhi* is described for the first time. In addition, the DNA barcode COI for the two species was obtained to estimate p-distance.

## Introduction

**Figure 1.**
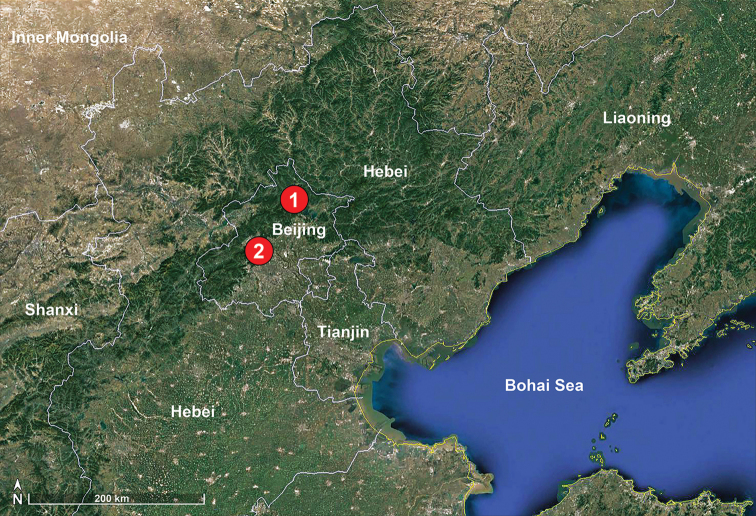
Distribution records of two *Pholcus* species **1***P. tongyaoi* sp. nov. **2***P. lexuancanhi* Yao, Pham & Li, 2012.

**Figure 2.**
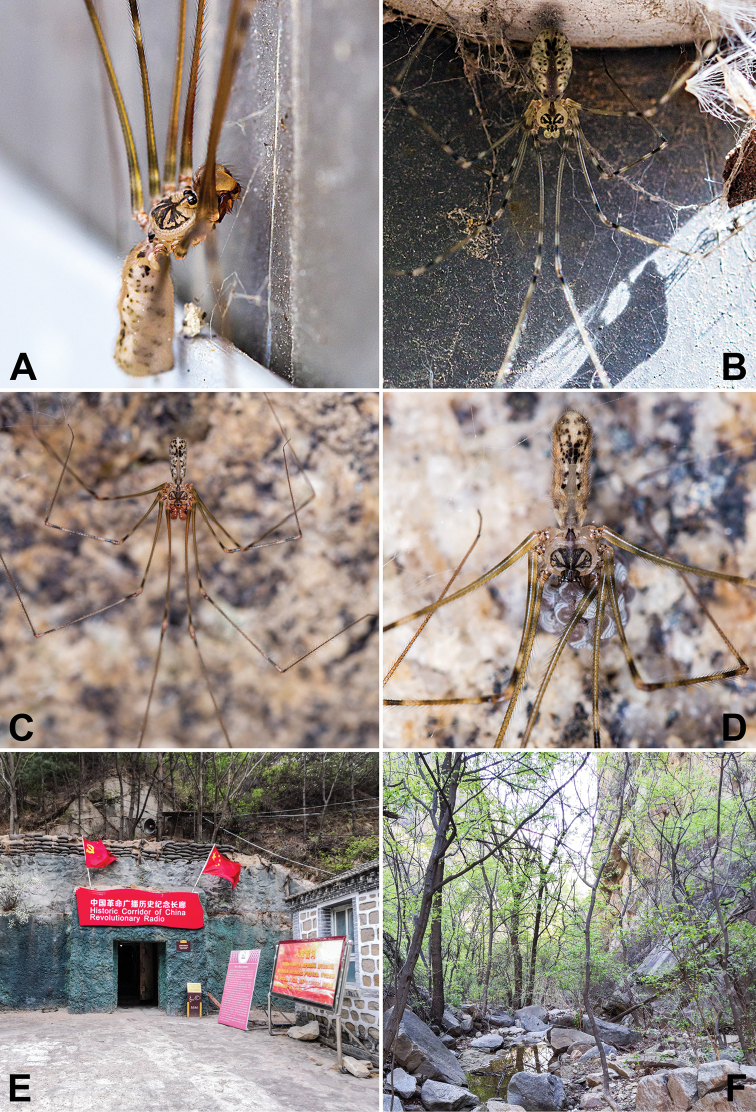
*Pholcus tongyaoi* sp. nov., live specimens and habitat **A, B** adult and juvenile males in old house **C, D** adult male and female with egg-sac on rock walls **E, F** habitat. Photographs by T Jiang (IZCAS).

The spider family Pholcidae C.L. Koch, 1850 contains 94 genera and 1768 species (World Spider Catalog 2020). It is among the most species-rich families and has a worldwide distribution (World Spider Catalog 2020). It is composed of five subfamilies: Ninetinae Simon, 1890, Arteminae Simon, 1893, Modisiminae Simon, 1893, Smeringopinae Simon, 1893, and Pholcinae C.L. Koch, 1850 based on recent morphological and molecular phylogenetic analyses (Huber 2011a; Dimitrov et al. 2013; Eberle et al. 2018). Pholcid spiders occupy a wide range of habitats in a variety of ecosystems, e.g., in buildings, under rocks, in crevices, in caves, in leaf litter, and in webs between trunks and twigs of trees (Huber 2005; Yao and Li 2012). *Pholcus* Walckenaer, 1805 is the most diverse genus in Pholcinae and Pholcidae, with 338 described species mainly distributed in the Palaearctic, Indo-Malayan, Afrotropical, and Australasian Region (Huber 2011b; Yao and Li 2012; World Spider Catalog 2020). These species belong to 21 species groups, of which the *Pholcus phungiformes* species group is highly diverse, including 53 known species definitively assigned to this species group (Huber 2011b; Peng and Zhang 2013; Kim and Ye 2015; Zhang et al. 2016; Huber et al. 2018; Zhu et al. 2018). The *P. phungiformes* species group is largely restricted to northeastern China and the Korean Peninsula; only *P. phungiformes* Oliger, 1983 occurs in Maritime Territory, Sakhalin Island, and Kurile Islands, Russia, probably as a result of human transport (Huber 2011b; World Spider Catalog 2020). This species group can often be found in caves, at cave entrances or on rock walls (Figs 1, 2), and diagnosed by the following characters: eight eyes, carapace with radiating marks, cylindrical opisthosoma, male chelicerae usually with frontal apophyses, male pedipalpal tibia with prolatero-ventral projection, procursus usually with dorsal spines, appendix absent, sometimes with ‘pseudo-appendix’, external female genitalia sclerotized, with knob (Huber 2011b; Zhu et al. 2018).

In this study, we describe one new species based on males and females from Huairou District, Beijing, China assigned to the *P. phungiformes* species group and redescribe its similar species *Pholcus lexuancanhi* Yao, Pham & Li, 2012 from a neighboring locality. The female of *P. lexuancanhi* is reported for the first time and the DNA barcode COI for the two species was obtained to estimate p-distance.

## Materials and methods

Specimens were examined and measured with a Leica M205 C stereomicroscope. Left male pedipalps were illustrated. External female genitalia were photographed before dissection. Vulvae were previously treated in a 10% warm solution of potassium hydroxide (KOH) to dissolve soft tissues before illustration. Images were captured with a Canon EOS 750D wide zoom digital camera (24.2 megapixels) mounted on the stereomicroscope mentioned above, and assembled using Helicon Focus 3.10.3 image stacking software (Khmelik et al. 2005). All measurements are given in millimeters (mm). Leg measurements are shown as: total length (femur + patella + tibia + metatarsus + tarsus), missing data were coded as ‘–’. Leg podomeres were measured on their dorsal side. The distribution map was generated with Google Earth Pro 7.3.2 (Google Limited Liability Company). The specimens studied are preserved in 75% ethanol and deposited in the College of Life Science, Shenyang Normal University (**SYNU**) in Liaoning, China and the Institute of Zoology, Chinese Academy of Sciences (**IZCAS**) in Beijing, China.

Terminology and taxonomic descriptions follow Huber (2011b) and Yao et al. (2015). The following abbreviations are used in the descriptions:

**ALE** anterior lateral eye,

**AME** anterior median eye,

**PME** posterior median eye,

**L/d** length/diameter.

DNA barcode was obtained for estimation of p-distance between *P. tongyaoi* sp. nov. and *P. lexuancanhi*. A partial fragment of the mitochondrial cytochrome oxidase subunit I (COI) gene was amplified and sequenced, using the following primers: forward: LCO1490-oono (5’-CWACAAAYCATARRGATATTGG-3’) and reverse: C1-N-2776 (5’-GGATAATCAGAATANCGNCGAGG-3’). DNA sample is preserved in TE buffer and stored at -20 °C. The sequences are deposited in GenBank. COI p-distance is computed with MEGA 5 (Tamura et al. 2011). For additional information on extraction, amplification, and sequencing procedures, see Yao et al. (2016).

## Taxonomic accounts

### Family Pholcidae C.L. Koch, 1850

**Subfamily Pholcinae C.L. Koch, 1850**

#### Genus Pholcus

Taxon classificationAnimaliaAraneaePholcidae

Walckenaer, 1805

8B61DDBF-FC36-514F-9625-A6BE00F129EC

##### Type species.

*Aranea phalangioides* Fuesslin, 1775.

#### Pholcus phungiformes

Taxon classificationAnimaliaAraneaePholcidae

species group

8F04BF74-7F1E-532D-A52D-BEDDDC5A2110

##### Diagnosis and description.

See Huber (2011b).

##### Remarks.

The ‘appendix’ in the original figures of four species apparently arises from the uncus: *P. papilionis* Peng & Zhang, 2011, *P. chiakensis* Seo, 2014, *P. gajiensis* Seo, 2014, and *P. palgongensis* Seo, 2014. We consider this a divided ‘pseudo-appendix’ and assign them to the *P. phungiformes* species group. Moreover, although the species *P. xianrendong* Liu & Tong, 2015 does not possess a prolatero-ventral projection on the male pedipalpal tibia, the bulb without appendix, the locality of this species is within the range of the *P. phungiformes* species group. Therefore, we tentatively assigned *P. xianrendong* to this species group. In total, this species group now contains 59 species. Of these, one species is newly described below.

#### Pholcus tongyaoi

Taxon classificationAnimaliaAraneaePholcidae

Wang & Yao sp. nov.

0B8D0243-D3A4-5187-B0D0-0681B4D522DC

http://zoobank.org/F7249E28-D367-4CA4-9D17-32977C5E5345

Figs 3 , 4

##### Type material.

***Holotype***: male (SYNU-Ar00016), Pool and Valley Natural Park (40°32.600'N, 116°40.687'E, elevation 574 m), Huairou District, **Beijing**, **China**, 26 April 2019, T Jiang leg. ***Paratypes***: 2 males (SYNU-Ar00017, Ar00018, GenBank number in SYNU-Ar00017: MT843113), same data as holotype; 2 females (SYNU-Ar00019, Ar00020), same data as holotype but 23 April 2019.

##### Etymology.

The specific name is a patronym in honor of the collector Tongyao Jiang (IZCAS) and is a noun (name) in genitive case.

##### Diagnosis.

**Figure 3.**
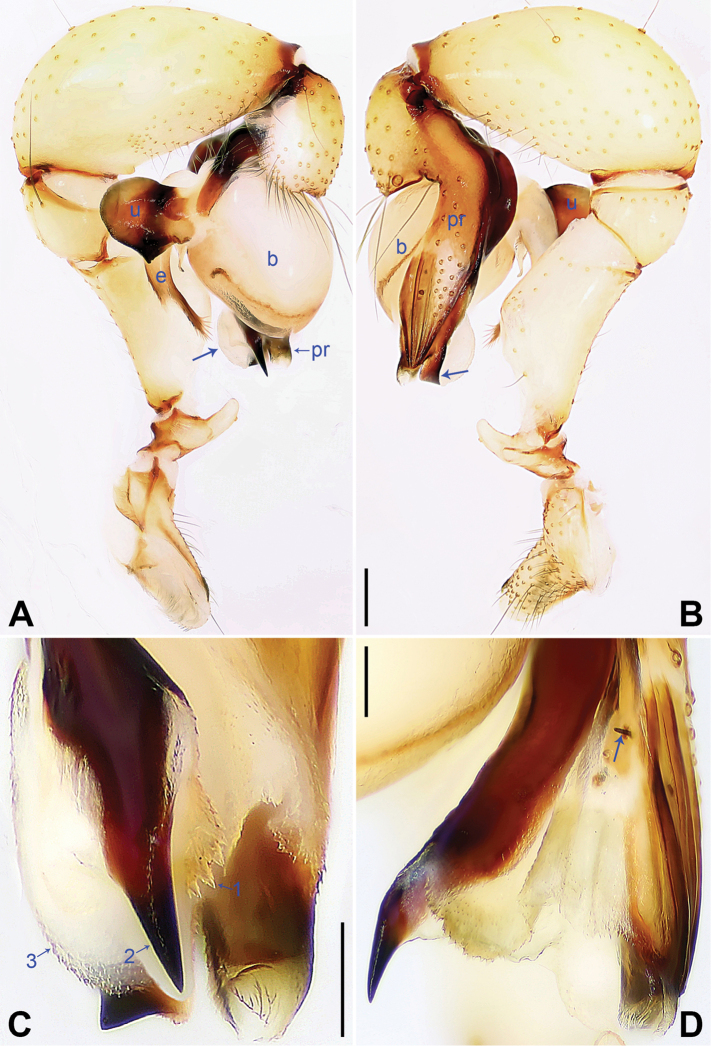
*Pholcus tongyaoi* sp. nov., holotype (**A, B, D**) and paratype (**C**) males **A, B** pedipalp (**A** prolateral view, arrow indicates ventral membranous process **B** retrolateral view, arrow indicates ventral sclerite) **C, D** distal part of procursus (**C** prolateral view, arrow 1 indicates prolateral membranous lamella, arrow 2 indicates spine-shaped prolateral apophysis, arrow 3 indicates ventral membranous process **D** dorsal view, arrow indicates dorsal spine). Abbreviations: b = bulb, e = embolus, pr = procursus, u = uncus. Scale bars: 0.20 (**A, B**), 0.10 (**C, D**).

**Figure 4.**
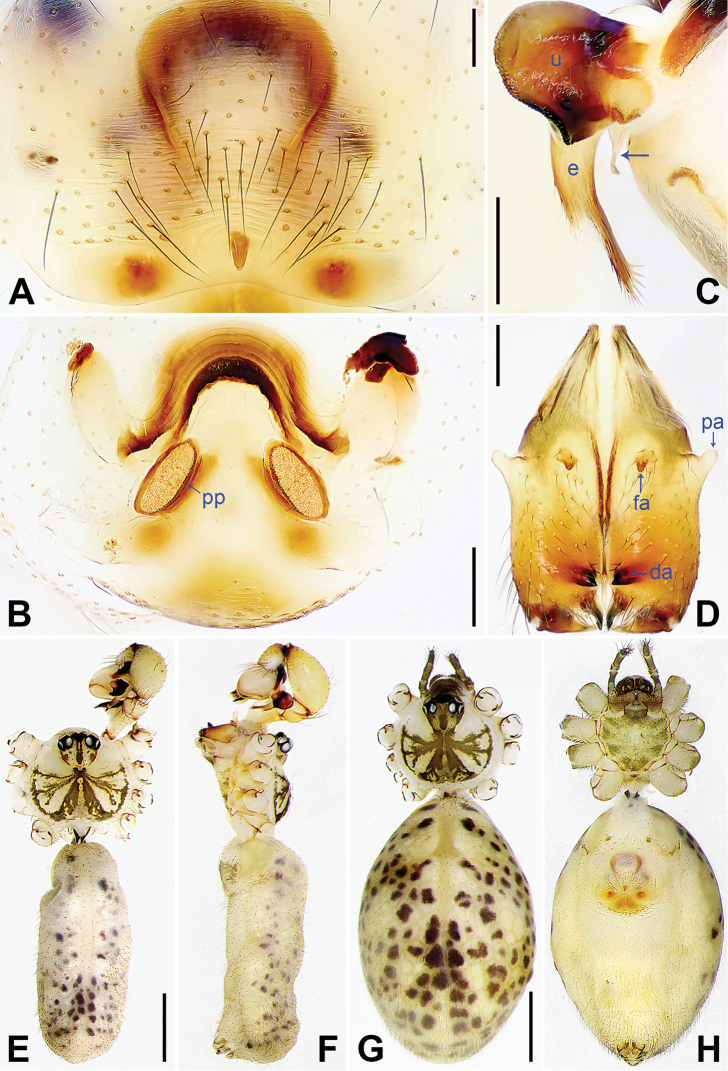
*Pholcus tongyaoi* sp. nov., holotype male (**C–F**) and paratype female (**A, B, G, H**) **A** external female genitalia, ventral view **B** vulva, dorsal view **C** bulbal apophyses, prolateral view, arrow indicates ‘pseudo-appendix’ **D** chelicerae, frontal view **E–H** habitus (**E, G** dorsal view **F** lateral view **H** ventral view). Abbreviations: da = distal apophysis, e = embolus, fa = frontal apophysis, pa = proximo-lateral apophysis, pp = pore plate, u = uncus. Scale bars: 0.20 (**A–D**), 1.00 (**E–H**).

**Figure 5.**
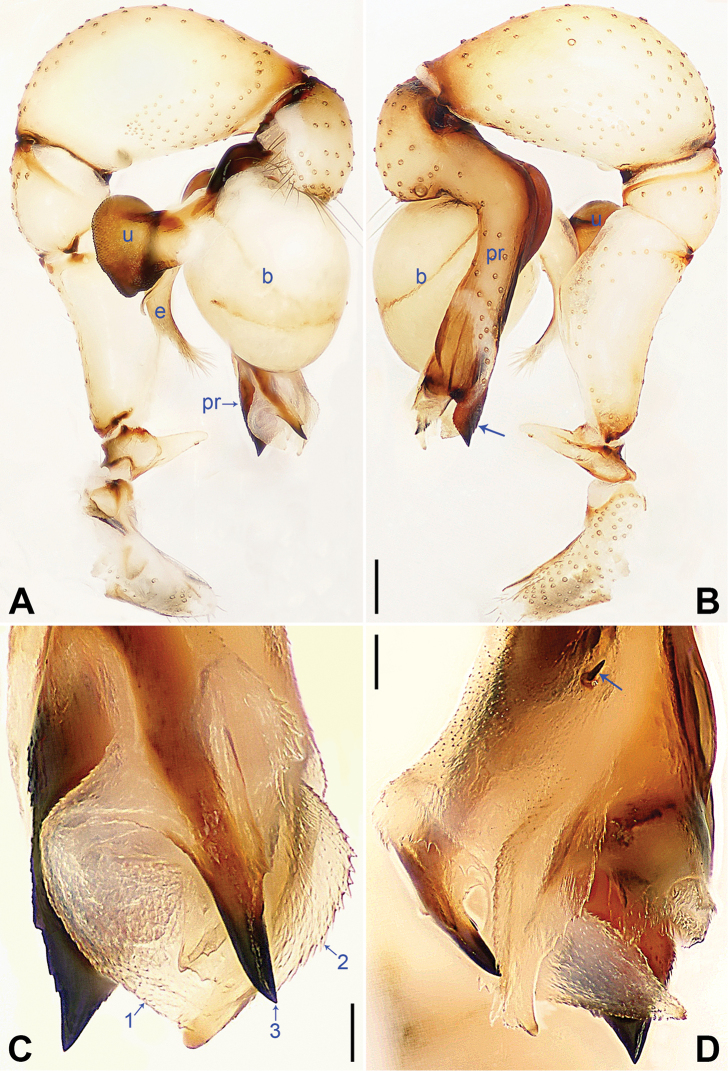
*Pholcus lexuancanhi* Yao, Pham & Li, 2012, male **A, B** pedipalp (**A** prolateral view **B** retrolateral view, arrow indicates ventral sclerite) **C, D** distal part of procursus (**C** prolateral view, arrows 1 and 2 indicate prolateral and dorsal membranous lamella, respectively, arrow 3 indicates spine-shaped prolateral apophysis **D** dorsal view, arrow indicates dorsal spine). Abbreviations: b = bulb, e = embolus, pr = procursus, u = uncus. Scale bars: 0.20 (**A, B**), 0.05 (**C, D**).

**Figure 6.**
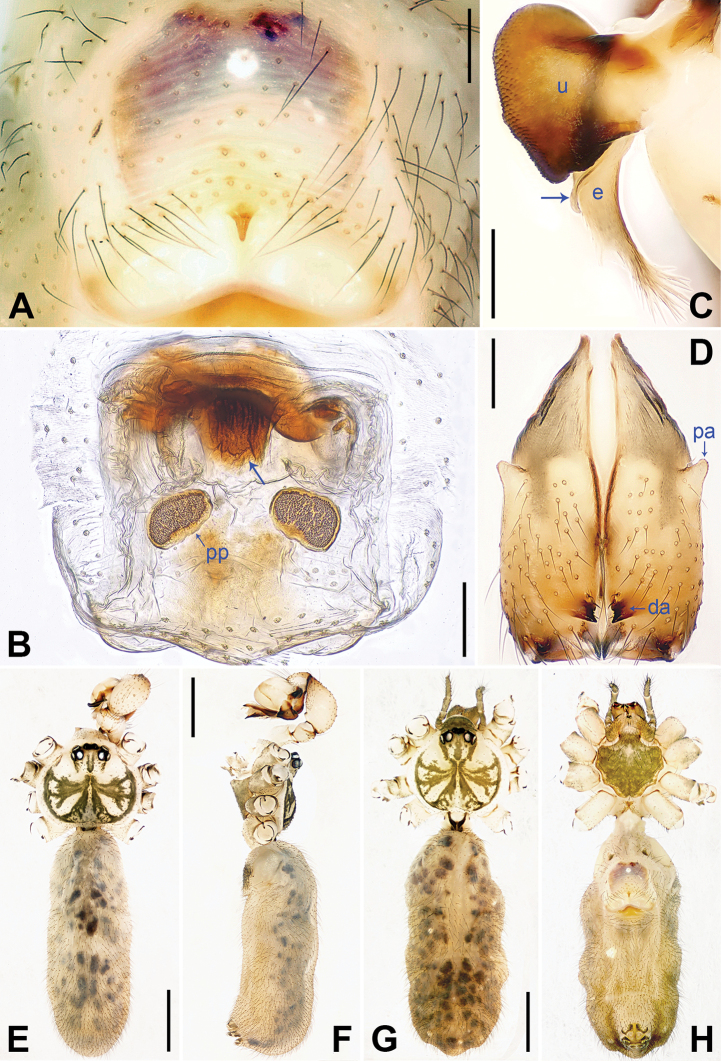
*Pholcus lexuancanhi* Yao, Pham & Li, 2012, male (**C–F**) and female (**A, B, G, H**) **A** external female genitalia, ventral view **B** vulva, dorsal view, arrow indicates median sclerite of anterior arch **C** bulbal apophyses, prolateral view, arrow indicates ‘pseudo-appendix’ **D** chelicerae, frontal view **E–H** habitus (**E, G** dorsal view **F** lateral view **H** ventral view). Abbreviations: da = distal apophysis, e = embolus, pa = proximo-lateral apophysis, pp = pore plate, u = uncus. Scale bars: 0.20 (**A–D**), 1.00 (**E–H**).

The species resembles *P. lexuancanhi* Yao, Pham & Li, 2012 (Figs 5, 6; Yao et al. 2012: 313, figs 1–15) with similar bulbal apophyses (Fig. 4C) and external female genitalia (Fig. 4A), but can be easily distinguished by procursus with large, semicircular, ventral membranous process (arrowed in Fig. 3A; prolateral membranous lamella in *P. lexuancanhi*, arrowed 1 in Fig. 5C), small, prolateral membranous lamella provided with sawtooth (arrowed 1 in Fig. 3C; large, dorsal membranous lamella in *P. lexuancanhi*, arrowed 2 in Fig. 5C), and small, angular ventral sclerite provided with curved tip (arrowed in Fig. 3B; large ventral sclerite and its tip not curved in *P. lexuancanhi*, arrowed in Fig. 5B), by male chelicerae with pair of frontal apophyses (arrowed fa in Fig. 4D; absent in *P. lexuancanhi*, Fig. 6D), and by vulva with n-shaped anterior arch without median sclerite (Fig. 4B; slightly curved anterior arch with median sclerite in *P. lexuancanhi*, arrowed in Fig. 6B) and elliptic pore plates (Fig. 4B; oval in *P. lexuancanhi*, Fig. 6B). This species can also be distinguished from *P. lexuancanhi* by COI p-distance 0.106 between them.

##### Description.

**Male** (**holotype**, SYNU-Ar00016): Total length 4.75 (4.93 with clypeus), carapace 1.56 long, 1.75 wide, opisthosoma 3.19 long, 1.34 wide. Leg I: – (11.62 + 0.75 + – + – + –), leg II: 30.89 (8.50 + 0.55 + 7.84 + 12.75 + 1.25), leg III: 20.62 (6.40 + 0.60 + 5.12 + 7.60 + 0.90), leg IV: 28.58 (8.40 + 0.62 + 7.12 + 11.12 + 1.32). Distance PME-PME 0.20, diameter PME 0.12, distance PME-ALE 0.05, distance AME-AME 0.02, diameter AME 0.08. Sternum wider than long (1.04/0.96). Habitus as in Fig. 4E, F. Carapace yellowish, with brown radiating marks and marginal brown bands; ocular area yellowish, with median and lateral brown bands; clypeus yellowish; sternum yellowish, with marginal brown marks. Legs yellowish, but dark brown on patellae and whitish on distal parts of femora and tibiae, with darker rings on subdistal parts of femora and proximal and subdistal parts of tibiae. Opisthosoma yellowish, with dorsal and lateral spots. Ocular area elevated, without eye stalks. Thoracic furrow absent. Chelicerae (Fig. 4D) with pair of proximo-lateral apophyses, pair of distal apophyses provided with two teeth each, and pair of frontal apophyses. Pedipalps as in Fig. 3A, B; trochanter with long, retrolaterally strongly bulged ventral apophysis; femur with indistinct ventral protuberance; tibia with prolatero-ventral projection; procursus simple proximally but complex distally, with large, semicircular, ventral membranous process (arrowed in Fig. 3A), small, prolateral membranous lamella with sawtooth (arrowed 1 in Fig. 3C), small, angular ventral sclerite with curved tip (arrowed in Fig. 3B), spine-shaped prolateral apophysis (arrowed 2 in Fig. 3C), and dorsal spine (arrowed in Fig. 3D); bulb with short curved ‘pseudo-appendix’ (arrowed in Fig. 4C); uncus with scaly edge (Fig. 4C); embolus weakly sclerotized, with some transparent distal projections (Fig. 4C). Legs with short vertical setae on tibiae, metatarsi, and tarsi, without spines or curved setae.

**Female** (**paratype**, SYNU-Ar00019): Similar to male, habitus as in Fig. 4G, H. Total length 5.31 (5.56 with clypeus), carapace 1.43 long, 1.14 wide, opisthosoma 3.88 long, 2.43 wide; tibia I: 5.90; tibia I L/d: 54. Distance PME-PME 0.18, diameter PME 0.12, distance PME-ALE 0.04, distance AME-AME 0.03, diameter AME 0.08. Sternum wider than long (1.07/0.83). Clypeus brown. External female genitalia (Fig. 4A) curved posteriorly, with short knob. Vulva (Fig. 4B) with sclerotized, n-shaped anterior arch and pair of elliptic pore plates. Retrolateral trichobothrium of tibia I at 4% proximally; tarsus I with 22 distinct pseudosegments.

##### Variations.

Ventral membranous process on procursus nearly crescent-shaped (arrowed 3 in Fig. 3C) in one paratype male (SYNU-Ar00017). Leg I missing in two paratype males (SYNU-Ar00017, Ar00018), total length 5.36 (5.52 with clypeus) in SYNU-Ar00017, total length 4.50 (4.60 with clypeus) in SYNU-Ar00018. Tibia I in another paratype female (SYNU-Ar00020): 5.75.

##### Distribution.

China (Beijing, type locality; Fig. 1).

##### Natural history.

The species was found in an old house and on rock walls.

#### Pholcus lexuancanhi

Taxon classificationAnimaliaAraneaePholcidae

Yao, Pham & Li, 2012

25AA5C3D-6173-5EB3-A001-514588A39779

Figs 5 , 6

Pholcus lexuancanhi Yao et al. 2012: 313, figs 1–15. Yao et al. 2015: 15.

##### Material examined.

2 males (IZCAS-Ar40901, Ar40902, GenBank number in IZCAS-Ar40901: MT843112) and 2 females (IZCAS-Ar40903, Ar40904), Beijing Botanical Garden (40°00'N, 116°12'E; type locality), Haidian District, **Beijing**, **China**, 30 July 2017, Z Yao leg.

##### Diagnosis.

See diagnosis for *P. tongyaoi* sp. nov.

##### Redescription.

**Male** (IZCAS-Ar40901): Total length 5.13 (5.38 with clypeus), carapace 1.41 long, 1.75 wide, opisthosoma 3.72 long, 1.44 wide. Leg I: 47.97 (14.47 + 0.80 + 11.41 + 18.53 + 2.76), leg II: 30.06 (8.46 + 0.78 + 7.24 + 11.79 + 1.79), leg III: 21.37 (6.35 + 0.59 + 5.13 + 8.08 + 1.22), leg IV: 28.03 (8.33 + 0.78 + 7.05 + 10.51 + 1.36); tibia I L/d: 76. Distance PME-PME 0.25, diameter PME 0.10, distance PME-ALE 0.05, distance AME-AME 0.05, diameter AME 0.10. Sternum wider than long (1.05/0.88). Habitus as in Fig. 6E, F. Carapace yellowish, with brown radiating marks and marginal brown bands; ocular area yellowish, with median and lateral brown bands; clypeus yellowish; sternum brown. Legs yellowish, but dark brown on patellae and whitish on distal parts of femora and tibiae, with darker rings on subdistal parts of femora and proximal and subdistal parts of tibiae. Opisthosoma yellowish, with dorsal and lateral spots. Ocular area elevated, without eye stalks. Thoracic furrow absent. Chelicerae (Fig. 6D) with pair of proximo-lateral apophyses and pair of distal apophyses provided with two teeth each. Pedipalps as in Fig. 5A, B; trochanter with long, retrolaterally strongly bulged ventral apophysis; femur with indistinct ventral protuberance; tibia with prolatero-ventral projection; procursus simple proximally but complex distally, with large, prolateral membranous lamella (arrowed 1 in Fig. 5C), large, dorsal membranous lamella with sawtooth (arrowed 2 in Fig. 5C), large, angular ventral sclerite (arrowed in Fig. 5B), spine-shaped prolateral apophysis (arrowed 3 in Fig. 5C), and dorsal spine (arrowed in Fig. 5D); bulb with short curved ‘pseudo-appendix’ (arrowed in Fig. 6C); uncus with scaly edge (Fig. 6C); embolus weakly sclerotized, with some transparent distal projections (Fig. 6C). Retrolateral trichobothrium of tibia I at 5% proximally; legs with short vertical setae on tibiae, metatarsi, and tarsi, without spines or curved setae; tarsus I with 33 distinct pseudosegments.

**Female** (IZCAS-Ar40903): Similar to male, habitus as in Fig. 6G, H. Total length 5.19 (5.38 with clypeus), carapace 1.36 long, 1.66 wide, opisthosoma 3.83 long, 1.68 wide; tibia I: 8.50; tibia I L/d: 54. Distance PME-PME 0.20, diameter PME 0.10, distance PME-ALE 0.05, distance AME-AME 0.05, diameter AME 0.08. Sternum wider than long (1.08/0.92). Clypeus brown. External female genitalia (Fig. 6A) curved posteriorly, with short knob. Vulva (Fig. 6B) with slightly curved, sclerotized anterior arch provided with median sclerite (arrowed in Fig. 6B) and pair of oval pore plates.

##### Variations.

Tibia I in another male (IZCAS-Ar40902): 11.54. Tibia I in another female (IZCAS-Ar40904): 9.12.

##### Distribution.

China (Beijing, type locality; Fig. 1).

##### Natural history.

The species was found on rock walls.

## Discussion

The *P. phungiformes* species group is highly diverse and currently contains 59 species including one new species in this study. These species are mainly distributed in three large mountain ranges (see the checklist below): the Mountain Taihang from southern North China (22 spp.), the Mountain Changbai (also called Mountain Paekdu in North Korea) from the border between northeastern China and North Korea (15 spp., of which *P. phungiformes* also occurs further east), and the Mountain Taebaek from Korean Peninsula (22 spp.) (Huber 2011b; Peng and Zhang 2011; Yao and Li 2012; Yao et al. 2012; Peng and Zhang 2013; Seo 2014; Kim and Ye 2015; Liu and Tong 2015; Zhang et al. 2016; Zhu et al. 2018). Nevertheless, the survey of the *P. phungiformes* species group is uneven. The highest diversity (43 spp.) concentrates in the Mountain Taihang and the southern Mountain Taebaek (South Korea). In contrast, only 15 species from the Mountain Changbai and one species from the northern Mountain Taebaek (North Korea) are recorded. Based on the high diversity of this species group from the southern Mountain Taebaek and the Mountain Taihang, as well as the similar landforms and habitats in neighboring northern Mountain Taebaek and Mountain Changbai, we strongly believe that additional species diversity likely remains undiscovered in the neighboring areas (e.g., Li 2020). Further survey in these areas is needed to fully understand the diversity that exists within this group.

A checklist of the *P. phungiformes* species group from three large mountain ranges is provided (for the complete list of references, see World Spider Catalog 2020):

The Mountain Taihang:

1. *Pholcus alloctospilus* Zhu & Gong, 1991

2. *Pholcus auricularis* Zhang, Zhang & Liu, 2016

3. *Pholcus babao* Tong & Li, 2010

4. *Pholcus beijingensis* Zhu & Song, 1999

5. *Pholcus brevis* Yao & Li, 2012

6. *Pholcus chicheng* Tong & Li, 2010

7. *Pholcus clavimaculatus* Zhu & Song, 1999

8. *Pholcus curvus* Zhang, Zhang & Liu, 2016

9. *Pholcus datan* Tong & Li, 2010

10. *Pholcus exilis* Tong & Li, 2010

11. *Pholcus jinniu* Tong & Li, 2010

12. *Pholcus lexuancanhi* Yao, Pham & Li, 2012

13. *Pholcus luya* Peng & Zhang, 2013

14. *Pholcus papilionis* Peng & Zhang, 2011

15. *Pholcus papillatus* Zhang, Zhang & Liu, 2016

16. *Pholcus pennatus* Zhang, Zhu & Song, 2005

17. *Pholcus suizhongicus* Zhu & Song, 1999

18. *Pholcus tongyaoi* sp. nov.

19. *Pholcus triangulatus* Zhang & Zhang, 2000

20. *Pholcus wangxidong* Zhang & Zhu, 2009

21. *Pholcus wuling* Tong & Li, 2010

22. *Pholcus zhuolu* Zhang & Zhu, 2009

The Mountain Changbai:

1. *Pholcus decorus* Yao & Li, 2012

2. *Pholcus fengcheng* Zhang & Zhu, 2009

3. *Pholcus foliaceus* Peng & Zhang, 2013

4. *Pholcus gaoi* Song & Ren, 1994

5. *Pholcus hamatus* Tong & Ji, 2010

6. *Pholcus jiuwei* Tong & Ji, 2010

7. *Pholcus lingulatus* Gao, Gao & Zhu, 2002

8. *Pholcus ningan* Yao & Li, 2018

9. *Pholcus phoenixus* Zhang & Zhu, 2009

10. *Pholcus phungiformes* Oliger, 1983

11. *Pholcus sublingulatus* Zhang & Zhu, 2009

12. *Pholcus tongi* Yao & Li, 2012

13. *Pholcus wangi* Yao & Li, 2012

14. *Pholcus wangtian* Tong & Ji, 2010

15. *Pholcus xianrendong* Liu & Tong, 2015

The Mountain Taebaek:

1. *Pholcus acutulus* Paik, 1978

2. *Pholcus cheongogensis* Kim & Ye, 2015

3. *Pholcus chiakensis* Seo, 2014

4. *Pholcus crassus* Paik, 1978

5. *Pholcus extumidus* Paik, 1978

6. *Pholcus gajiensis* Seo, 2014

7. *Pholcus gosuensis* Kim & Lee, 2004

8. *Pholcus joreongensis* Seo, 2004

9. *Pholcus juwangensis* Seo, 2014

10. *Pholcus kwanaksanensis* Namkung & Kim, 1990

11. *Pholcus kwangkyosanensis* Kim & Park, 2009

12. *Pholcus montanus* Paik, 1978

13. *Pholcus nodong* Huber, 2011

14. *Pholcus okgye* Huber, 2011

15. *Pholcus palgongensis* Seo, 2014

16. *Pholcus parkyeonensis* Kim & Yoo, 2009

17. *Pholcus pojeonensis* Kim & Yoo, 2008

18. *Pholcus simbok* Huber, 2011

19. *Pholcus socheunensis* Paik, 1978

20. *Pholcus sokkrisanensis* Paik, 1978

21. *Pholcus woongil* Huber, 2011

22. *Pholcus yeongwol* Huber, 2011

## Supplementary Material

XML Treatment for Pholcus

XML Treatment for Pholcus phungiformes

XML Treatment for Pholcus tongyaoi

XML Treatment for Pholcus lexuancanhi
